# HOW OFTEN AND WHY DO PEOPLE MANAGE THEIR EMOTIONS?: EMOTION REGULATION IN HEALTHY AGING AND MILD COGNITIVE IMPAIRMENT

**DOI:** 10.1093/geroni/igad104.0111

**Published:** 2023-12-21

**Authors:** Tabea Springstein, Tammy English

**Affiliations:** Washington University in St. Louis, St. Louis, Missouri, United States; Washington University in St. Louis, St. Louis, Missouri, United States

## Abstract

Prominent theories of adult development suggest that individuals increasingly prioritize emotional goals and social relationships as they grow older. Accordingly, older adults are expected to invest more in maintaining their emotional well-being compared to younger adults. Prior work suggests that older adults may accomplish this goal by structuring their lives in ways that reduce the need to manage unwanted emotion. We tested the hypotheses that (1) older adults regulate their emotions less often in daily life compared to younger adults, and (2) when emotion regulation occurs, older adults are relatively more motivated by pro-hedonic and social concerns. Using experience sampling (7x/day for 9 days), we assessed whether emotion regulation frequency and motives differ between younger adults (N = 70), cognitively normal older adults (N = 88), and older adults with mild cognitive impairment (MCI; N = 60). We found that older adults with and without MCI regulated their emotions less frequently than younger adults, even when controlling for mean levels of positive and negative emotional experience. However, there were largely no group differences in emotion regulation motives (i.e., why people wanted to manage their emotions). Future work is needed to explore how age-related differences in life contexts might contribute to less need for emotion regulation in relatively older adults. Less frequent regulation could be beneficial in terms of helping older adults preserve their more limited cognitive resources. The findings regarding motives add to growing research on aging which suggest maintenance or similarity in many emotion regulation processes across adulthood.

